# Use of Predicted Behavior from Accelerometer Data Combined with GPS Data to Explore the Relationship between Dairy Cow Behavior and Pasture Characteristics

**DOI:** 10.3390/s20174741

**Published:** 2020-08-22

**Authors:** Lucile Riaboff, Sébastien Couvreur, Aurélien Madouasse, Marie Roig-Pons, Sébastien Aubin, Patrick Massabie, Alain Chauvin, Nicolas Bédère, Guy Plantier

**Affiliations:** 1 ESEOTech-LAUM, Ecole Supérieure d’Electronique de l’Ouest, 49000 Angers, France; sebastien.aubin@reseau.eseo.fr (S.A.); guy.plantier@eseo.fr (G.P.); 2 Terrena Innovation, 44150 Ancenis, France; marie.roig-pons@agroscope.admin.ch (M.R.-P.); patrick.massabie@idele.fr (P.M.); 3 URSE, Ecole Supérieure d’Agricultures, University Bretagne Loire, 49000 Angers, France; s.couvreur@groupe-esa.com (S.C.); nicolas.bedere@inrae.fr (N.B.); 4 INRAE, BIOEPAR, Oniris, 44307 Nantes, France; aurelien.madouasse@oniris-nantes.fr (A.M.); alain.chauvin@oniris-nantes.fr (A.C.)

**Keywords:** predicted behaviors, cow location, animal–environment interaction, three-dimensional accelerometer, Global Positioning System, agro-ecology

## Abstract

Our aim in this study was to investigate whether the behaviors of dairy cows on pasture, predicted with accelerometer data and combined with GPS data, can be used to better understand the relationship between behaviors and pasture characteristics. During spring 2018, 26 Holstein cows were equipped with a 3D-accelerometer and a GPS sensor fixed on a neck-collar for five days. The cows grazed alternatively in permanent and in temporary grasslands. The structural elements, soil moisture, slope and botanical characteristics were identified. Behaviors were predicted every 10 s from the accelerometer data and combined with the GPS data. The time-budgets expressed in each characterized zone of 8 m × 8 m were calculated. The relation between the time-budgets and pasture characteristics was explored with a linear mixed model. In the permanent grassland, dairy cows spent more time under a tree to ruminate (*p* < 0.001) and to rest (*p* < 0.001) and more time to graze in areas with *Holcus lanatus* (*p* < 0.001). In the temporary grassland, behavior was influenced by the external environment (presence of other animals on the farm; *p* < 0.05). Thus, this methodology seems relevant to better understand the relationship between the behaviors of dairy cows and grazing conditions to develop precision grazing.

## 1. Introduction

Agro-ecology aims to develop practices to improve animal health and welfare and reduce farming environmental impacts while increasing farm profitability [1]. A better understanding of the interactions between the behavior expressed by ruminants on pasture and their environment, including vegetation, structural characteristics, soil moisture, etc., could be a way of making progress towards these objectives. As explained by Carvalho et al. [2], monitoring ruminant behavior is a way to achieve optimal plant production, animal forage intake and performances through a better understanding of how animals meet their requirements by grazing a dynamic vegetation. Investigating individual behaviors in relation to the environment could also help in targeting preventive treatments based on the exposure to specific risks [3]. Indeed, ruminants at pasture are more likely to develop certain diseases depending on the areas used [4,5]. A fine knowledge of the amount of time spent in areas associated with an increased risk of disease by individual animals could help to identify animals at risk and treat them specifically. Identifying over-used areas could also reduce the environmental impact at the farm level. Important amounts of cow excreta on small areas may lead to localized releases of nitrogen into the soil. Identifying such areas could help to adapt management practices accordingly [6]. Finally, focusing on the relationship between ruminants and their environment is probably an interesting way to explore changes of behavior due to challenging situations [7]. For example, Putfarken et al. [8] showed that cows preferentially grazed close to drinking troughs when the temperature was high, suggesting that a fine understanding of the relationship between animals and their environment should help to identify patterns of discomfort at grazing.

Such an investigation requires continuous and automatic information on the position and behavior of ruminants, i.e., with a high frequency and without human intervention. The Global Positioning System (GPS) has already been used both to predict the behavior of ruminants [9] and to geolocalize them in pastures [8]. Although the GPS does not seem appropriate for predicting behaviors in a robust way [10], this geolocation system seems promising for locating animals with a high frequency and with a low error (3.9 m ± 0.8 m standard error with uncorrected data [11]). Moreover, Putfarken et al. [8] fixed GPS sensors on cattle and sheep to elucidate which vegetation types are preferentially used and whether animal preferences change through the seasons in semi-open cultural landscapes. However, the behaviors expressed on trajectories were not known in this study. Three-dimensional accelerometer sensors have also already been used to predict and collect states (standing, lying, walking, etc.) [12] or unitary behaviors (biting, chewing) [13] with a high frequency and an accuracy higher than 90%. Although the range of correctly predicted behaviors is not sufficient in these studies to explore finely the interactions between animals and their environment, the combination of (i) predicted behaviors from accelerometer data with (ii) the corresponding position of ruminants on the pasture collected with GPS sensors is certainly a promising approach to explore the interactions between ruminants and their environment.

In two previous studies, a methodological framework was developed to predict a large range of behaviors of dairy cows on pasture from accelerometer data every 10 s, including grazing, walking, resting and ruminating both in lying and standing postures [14,15]. These predicted behaviors could now be combined with the position of the cows using the GPS data collected on animals. In this study, we propose to investigate whether such a combination could be used to relate the expressed behaviors at grazing to the pasture characteristics. 

## 2. Materials and Methods

**Figure 1:**
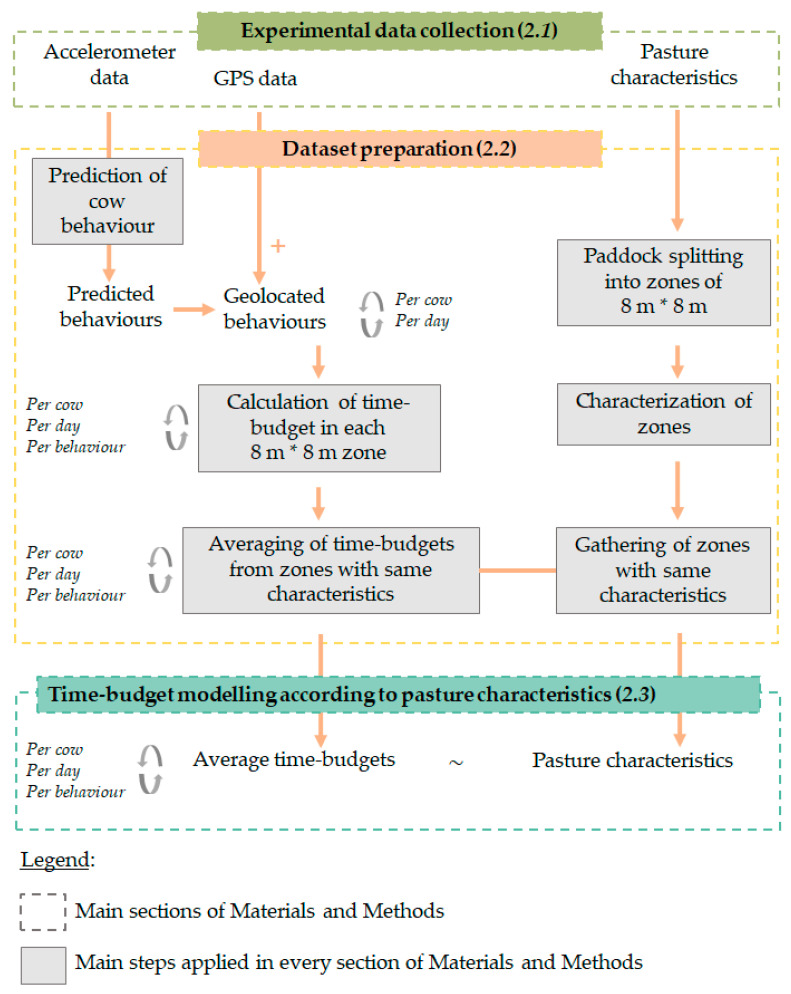
Overview of the main steps applied in the Material and Methods section.

An overview of the applied methodological framework is provided in Figure 1.

### 2.1. Experimental Design

#### 2.1.1. Farm, Animal and Sensors Description

Data collection was carried out in a 71 Holstein (milk yield: 10,000 kg per year) commercial farm in La Pommeraye, France (N 47°21′21″, O 0°51′33″) in May 2018. The average parity of the cows was 2.50 ± 1.40 (mean ± sd) and the average day in milk was 250 ± 167 (mean ± sd). Cows were milked using an automatic milking system (AMS) (milking count per day: 2.4 ± 0.7 (mean ± sd)) and they received a supplementation of triticale, peas and feverole (1.83 kg DM/cow) and a supplementation of feverole (0.13 kg DM/cow) after each AMS visit. During each grazing rotation, the 71 cows grazed in a first pasture from 05:00 h to 11:00 h and in a second one from 11:00 h to 18:00 h and from 21:00 h to 05:00 h. However, as cows (i) had to return to the barn to be milked and (ii) could stay in the barn instead of grazing, there were not always 71 cows on pasture at the same time. Cows were also held in the barn from 18:00 h to 21:00 h and received alfalfa forage (1.5 kg DM/cow) during this period. 

Twenty-six dairy cows were chosen to be representative of the herd in terms of parity and days in milk. During the experiment, the lactation stages ranged between 16 and 253 days in milk (153.7 ± 61.0 (mean ± sd)), with the parity ranging between one and seven (2.7 ± 1.6 (mean ± sd)). The parities and days in milk of the herd and of the selected cows at enrolment are provided in Appendix A (Table A1).

A RF-Track datalogger (RF-Track, Rennes, France) comprising a LSM9DS1 three-axis accelerometer (STMicroelectronics, Geneva, Switzerland) ±2 g and a GPS sensor (part number EVA-7M-0, µ-Blox, Thalwil, Switzerland) with a static position error estimated at ±1.72 m was used. Data were collected at 59.5 Hz and 1 Hz for the accelerometer data and GPS data, respectively. The sensors were powered with two 3.7 V lithium batteries (2.6 Ah). Data were stored on a secure digital card and downloaded after the experiment. The dataloggers were 98.2 mm × 51.60 mm× 36.0 mm in size and weighed 250 g. The dataloggers were fixed on a collar and positioned on the right side of the neck. The collars were tightly adjusted and a 500 g counterweight was added to prevent them from turning around. The *x*-axis detected the up–down direction, the *y*-axis detected the backward–forward direction and the *z*-axis detected the left–right direction. 

#### 2.1.2. Data Collection

##### Accelerometer and GPS Data 

Collars were mounted on the 26 chosen animals at 5:00 p.m. on 30 April 2018 and removed 5 days later at the same time, which corresponds to both the duration of the complete grazing rotation and the autonomy of the sensor battery. It should be noted that a period of habituation to the device by the cows was not necessary as the complete device represented approximately 0.1% of the animal’s weight [16]. The data collected from 21:00 h to 05:00 h were not used in this study to focus on the daytime behavior exclusively.

##### Weather

The temperatures ranged from 4 °C at the coldest time of day to 20 °C at the warmest time of day, except on the second and last day where the temperature dropped to 1.9 °C and reached 23.2 °C, respectively. The precipitation was less than 1 mm each day except for the second day where 3.2 mm were recorded. The wind speed was less than 40 km/h except on the second day (maximum gusts: 43.2 km/h). An average of 9.8 h of sunshine was recorded, with a minimum of 2.06 h on the second day and a maximum of 13.34 h on the last day [17].

##### Grass Height and Herbage Allowance

During the 5 days of the experiment, 71 dairy cows grazed in a first pasture of 1.6 ha from 05:00 h to 11:00 h and in a second one of 2.3 ha from 11:00 h to 18:00 h and from 21:00 h to 05:00 h. An average grass height of 15 cm was measured before the study period in each paddock based on 97 measures per ha using the GrassHopper plate meter [18]. Based on a usual density of 250 kg DM/ha/cm, the herbage mass was 2500 kg DM/ha > 5 cm. Considering a total area of 3.9 ha for 71 dairy cows during 5 days of grazing, the herbage allowance was therefore 27.5 kg DM/cow/day. 

##### Structural and Botanical Characteristics

**Figure 2:**
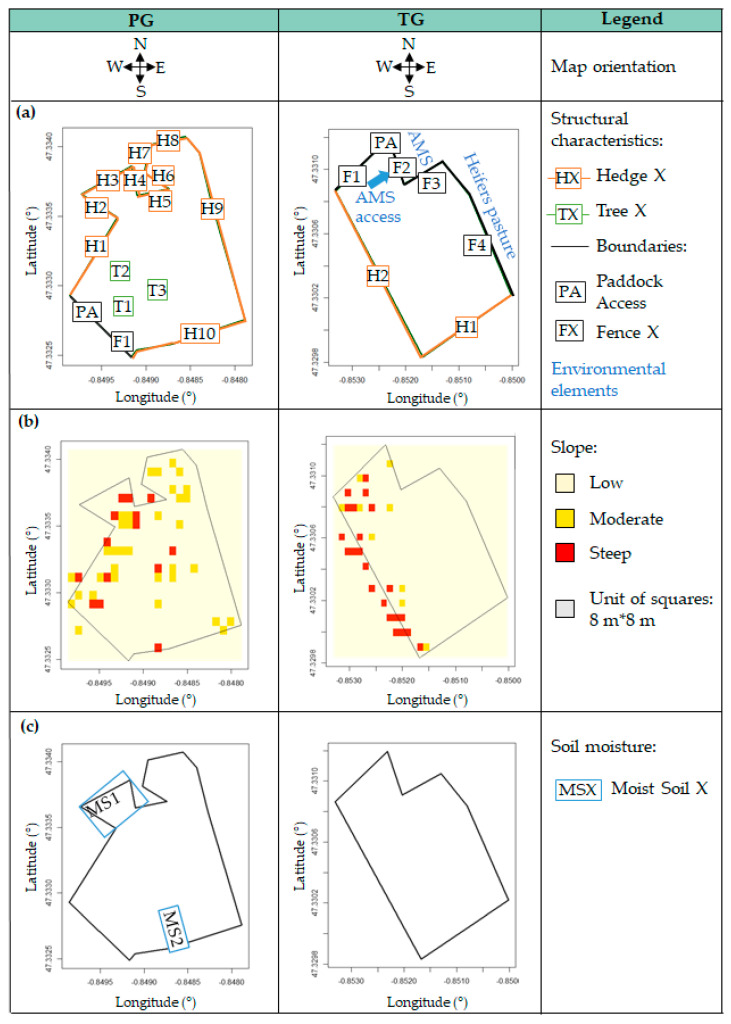
Description of the two paddocks grazed during the experiment based on the (**a**) structural characteristics (**b**) slope and (**c**) soil moisture.

The characteristics were collected in the paddocks grazed during the experiment. The first paddock was a permanent grassland of 1.6 ha (thereafter referred to PG). The second paddock was a temporary grassland of 2.3 ha (thereafter referred to TG). The locations of the structural elements, slope and moist soil areas are provided in Figure 2.

The pasture characteristics considered in each paddock are the following:

(i) The structural characteristics in the paddock including trees, hedges and boundaries were identified and geolocated (longitude and latitude) using the data from the French’s National Geographic Institute [19]. The locations of structural elements in each paddock are illustrated in Figure 2a.
(ii) The steepest slopes were located in the paddocks using the data from the French’s National Geographic Institute data [19]. The slopes ranging between 10% and 20% were referred to as a “moderate slope” and slopes higher than 20% were referred to as a “steep slope”. The remaining slopes were referred to as a “low slope” by default. Slopes in the paddocks are shown in Figure 2b.
(iii) The soil moisture was considered in the paddocks. Moist soil areas were located in the paddocks using the data from the French’s National Geographic Institute [19]. Such areas were referred to as “moist soil” and the remaining areas were referred to as “dry soil” by default. The soil moisture in the paddocks is provided in Figure 2c. It should be mentioned that no moist soil area was found in the TG.
(iv) The plant species were identified and recorded using a method based on the quadrat method [20,21]. Approximately 97 measures per hectare were carried out in each pasture, corresponding to 165 measures in the PG and 182 in the TG. For every measure, a rating ranging between 1 and 10 was attributed to the five most represented species in the area. The more a plant species was represented in the area, the closer its rating was to 10. The other plant species identified in the area were only noted without a rating. The bare ground was also considered in the rating. Seventy-six different plant species were identified in the PG and 41 in the TG. Each measure was also geolocated in the paddock based on the geolocation data obtained during grass height measurements with the GrassHopper plate meter (Section 2.1.2).

### 2.2. Dataset Preparation

The purpose of this step was to process the raw data in order to obtain the geolocalized predicted behavior from the accelerometer and GPS data, as well as the groups of homogeneous pasture patches from the different pasture characteristics collected. This step was carried out for both the PG and TG, separately. Data used to construct the dataset are available in Supplementary Materials.

#### 2.2.1. Prediction of Behaviors of Dairy Cows 

This step aimed to predict the behaviors successively expressed by the 26 dairy cows over the experiment. We used a method described in two previous studies which allows a prediction of the main behaviors of dairy cows on pasture from accelerometer data with a high reliability (accuracy: 98%; Cohen’s Kappa: 0.96) [14,15]. The six predicted behaviors are the following:

- Grazing: biting, taking frequent bites or chewing and searching without raising the head.
- Walking: movement from one location to another without lowering the head at ground level.
- Ruminating while lying: lying with regurgitating rumen bolus before chewing and then re-swallowing.
- Ruminating while standing: standing with regurgitating rumen bolus before chewing and then re-swallowing.
- Resting while lying: lying without rumination.
- Resting while standing: standing without movement or rumination.

**Figure 3:**
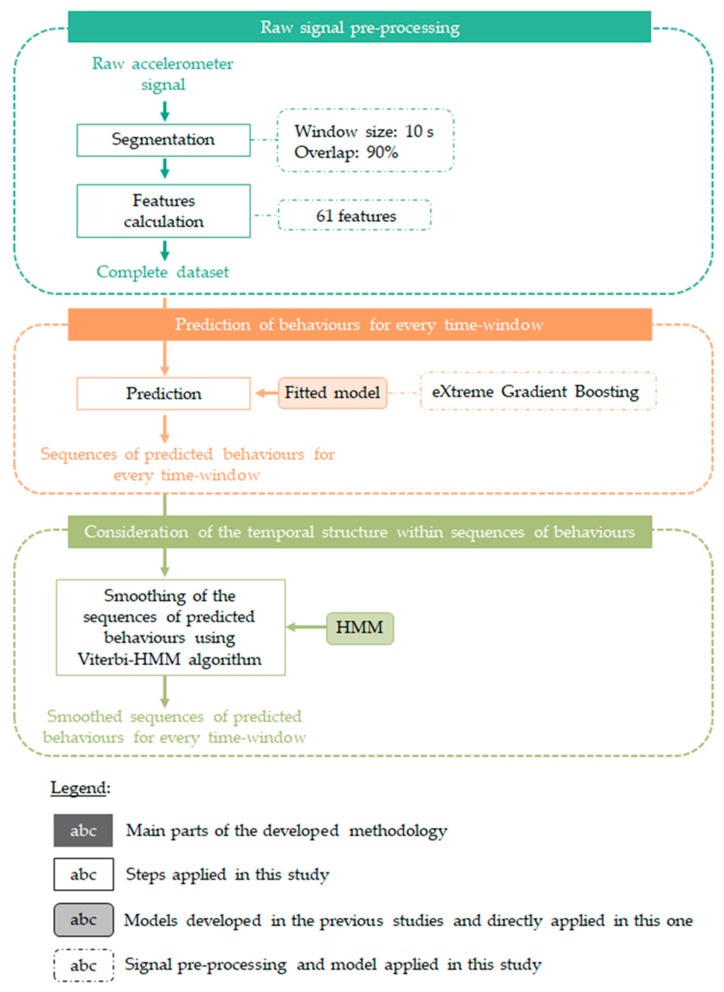
Description of the successive stages carried out in this study to obtain the predicted behaviors of dairy cows during the experiment.

**Figure A1:**
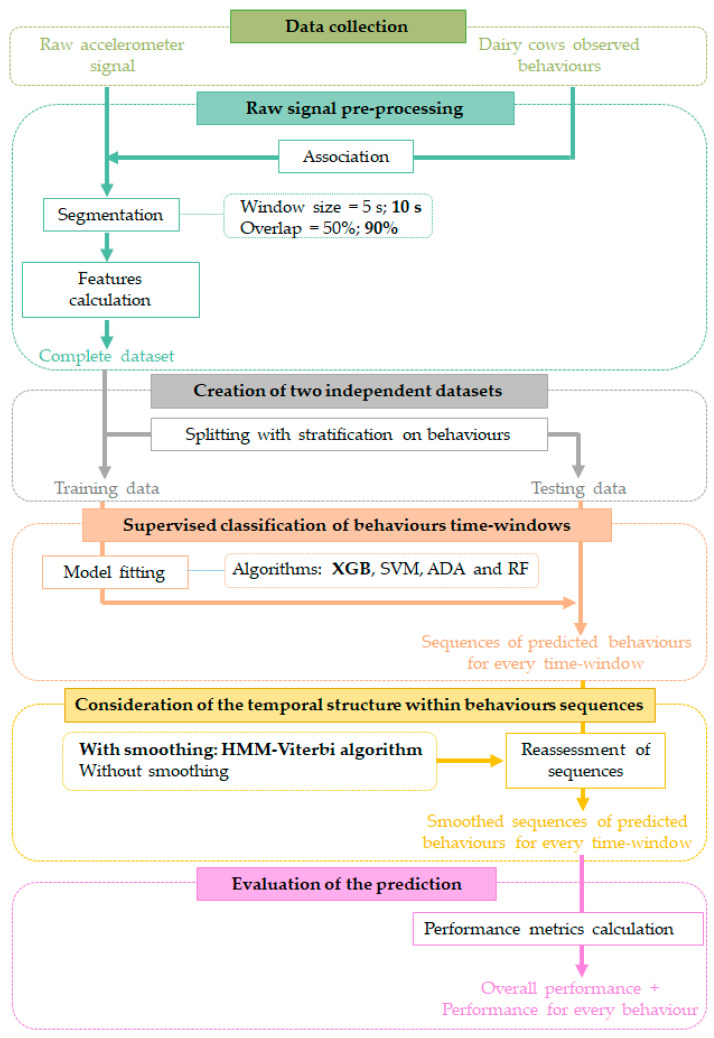
Method carried out in the previous studies to develop an appropriate methodology to predict the range of the main behaviors of dairy cows at pasture. Note: The techniques leading to the best performance (accuracy—98%; Cohen’s Kappa—0.96) are written in bold. The combination of the best techniques corresponds to the methodological framework used in the present study.

The steps carried out to develop this methodology are outlined in Appendix A (Figure A1) and we refer to Riaboff et al. [14,15] for a detailed description. The successive steps applied in the present study are illustrated in Figure 3. First, the raw accelerometer sequences collected from the 26 dairy cows during the 5 days of grazing were split into segments (windows) of 10 s, with 90% of the data being in common with two consecutive windows (overlap). This pre-processing step was performed in Matlab R2018a. The eXtreme Gradient Boosting (XGB) model fitted by Riaboff et al. [14] was then directly used to predict the behaviors using the xgboost package [22] in R 3.6.1 [23]. At the end of this step, the behavior of each cow throughout every successive window of 10 s was predicted for all 5 days of grazing. The predicted behaviors in successive windows from the same cow over the entire experiment were finally smoothed using the hidden Markov model (HMM)-based Viterbi algorithm [24,25] applied with the R package HMM [26]. The HMM used to apply the Viterbi algorithm in this study was reported by Riaboff et al. [14]. 

#### 2.2.2. Calculation of the Time-budget Expressed in Each Zone of the Pastures 

The aims of this step were to (1) collect the position of cows in pastures while they were expressing a behavior and (2) compute the overall cow location and time-budget for every behavior expressed in every zone in the pastures per cow and per day. 

The date and time associated to each record were collected for both the accelerometer and GPS data. The time synchronization between these two sensors was previously ensured. Each 10 s window of behavior was thus associated with the position (longitude and latitude) of the cow while she was expressing the behavior, based on the date and time of the accelerometer and GPS records. It should be noted that the GPS data were collected every second (1 Hz), therefore there were 10 recorded positions associated to each 10 s window. The longitude and latitude selected for the 10 s window were those corresponding to the mid-point recording within the window. The 10 s windows of behavior combined with the associated geographical coordinates were thereafter referred to as the geolocated behaviors.

**Figure 4:**
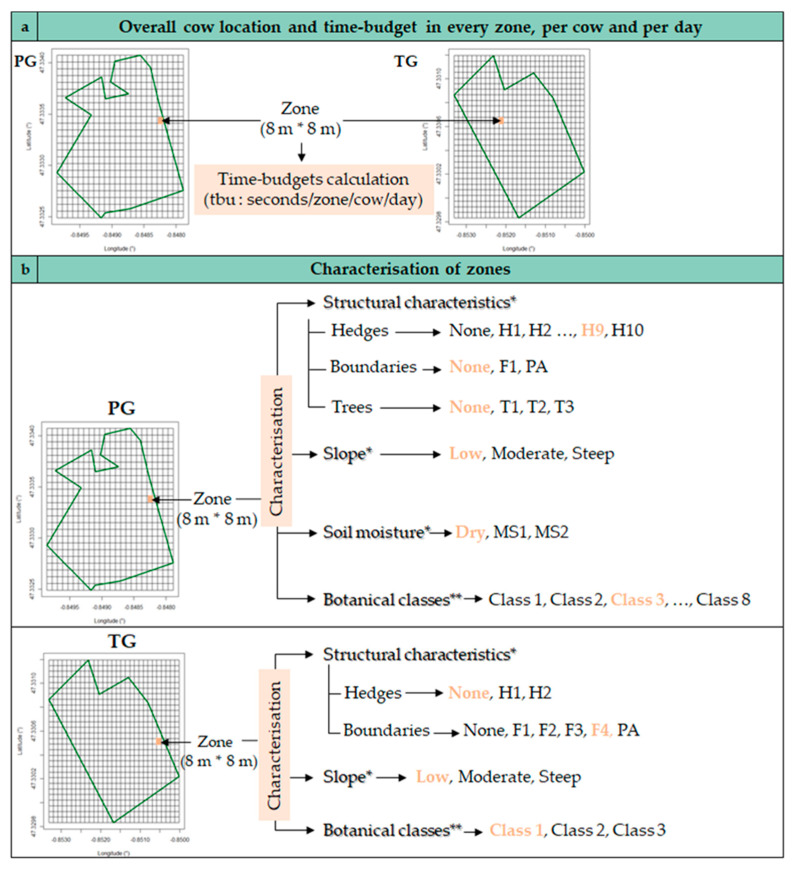
Time-budget computation in every zone, per cow and per day (**a**) and the characterization of the zones given the structural characteristics, slope, soil moisture and botanical classes (**b**) in the permanent grassland (PG) and temporary grassland (TG). Note: the complete characterization of the example grid zone marked orange on the map is given in orange in the adjacent text; * refer to the description provided in Figure 2; ** refer to the botanical classes provided in Figure 5.

Each paddock was split into squares of 8 m × 8 m, hereafter referred to as zones. This surface was chosen to broadly encompass the GPS static position error in the open area (±1.72 m) and to maintain a sufficiently fine description of the paddock. In this way, 576 zones and 841 zones were obtained in the PG and the TG, respectively. The geographical coordinates of each zone were recorded using the data from the French’s National Geographic Institute [19]. For every cow and every day of grazing, the longitude and latitude of the geolocated behaviors were used to identify in which 8 m × 8 m zone the cow was during the expression of the behavior, as illustrated in Figure 4a. 

This step was carried out using the R package sp [27]. The time-budgets associated with each behavior were therefore computed in each zone of the pastures for every cow and every day of grazing. The unit of these time-budgets (hereafter referred to as tbu) was thus seconds per area of 8 m × 8 m, per cow and per day. The overall cow location in each zone for every cow and for every day of grazing was the sum of the time-budgets in the corresponding zone. 

#### 2.2.3. Characterization of Each Zone in the Pasture

This step aimed to assign pasture characteristics to each area of each pasture. The pasture characteristics were defined by a combination of the structural characteristics, slope, soil moisture and botanical characteristics, as illustrated in Figure 4b. The characterization of zones was performed as follows using the sp package [27]:

- Structural characteristics

As explained in Section 2.1.2, the structural characteristics were geolocated in pastures (Figure 2a). The geographic coordinates were used to describe the structural characteristics in each zone. As illustrated in Figure 4b, each zone was described in terms of trees (T1, T2 or T3), hedges (H1, …, H10) and boundaries (F1 or PA) for the PG and in terms of hedges (H1 or H2) and boundaries (F1, F2, F3, F4 or PA) for the TG. By default, a zone was assigned to none if there was no structural characteristic nearby.

- Slope

As presented in Section 2.1.2., the steepest slopes were located in the pastures (Figure 2b). The geographic coordinates were used to assign a moderate slope or steep slope to the corresponding zones, as illustrated in the Figure 4b. By default, the remaining zones were assigned to low slope.

- Soil moisture

Areas with a moist soil were located in the PG as explained in Section 2.1.2. (Figure 2c). As illustrated in Figure 4b, two nonadjacent moist areas were identified in the PG and labelled MS1 and MS2. Otherwise, the zones were characterized as dry by default.

- Botanical characteristics

**Figure 5:**
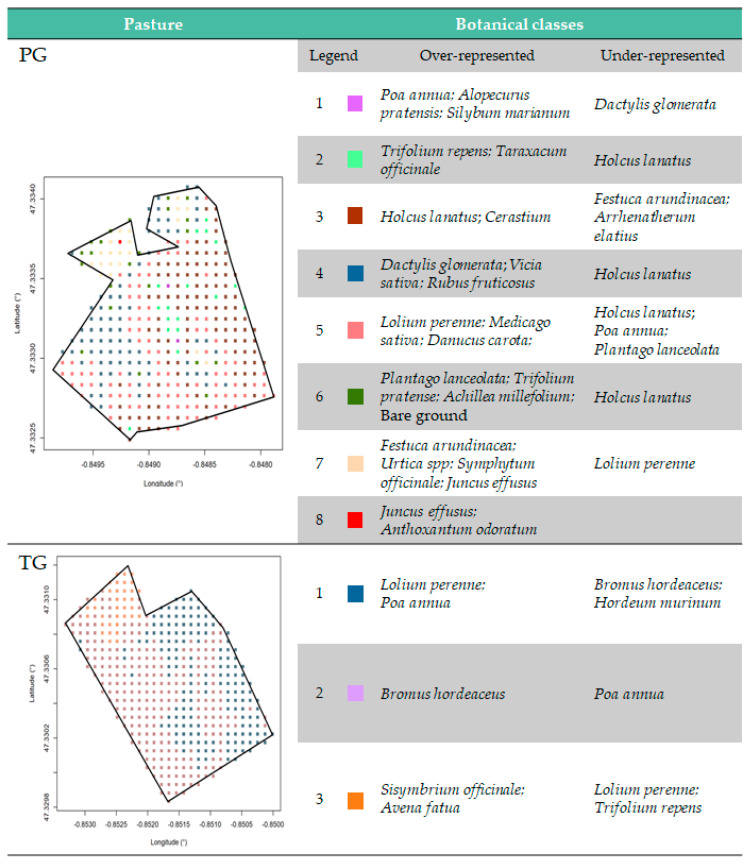
Description and geolocation of the botanical classes expressed with over-represented and under-represented plant species in the permanent grassland (PG) and temporary grassland (TG).

As explained in Section 2.1.2, plant species were identified in each pasture (97 measures/ha). As the number of different species identified in the pastures was very large (76 and 41 in the PG and TG, respectively), each geolocated measurement was described by a botanical class in a second stage, instead of keeping a rating for every species. For this purpose, a hierarchical ascendant classification (aggregation: Ward criterion) was applied with the R package FactoMiner [28]. Based on the evolution of intraclass inertia over the aggregation process, 8 and 3 classes were identified in the PG and TG, respectively. The botanical classes were then described by the plant species which are the most represented in the class (F-test: v-test > 2) and by the plant species which are the least represented in the class (F-test: v-test < −2). A spatial interpolation (krigeage) of botanical classes was carried out using the R packages gstat [29], rgdal [30] and raster [31] to get a botanical class in each zone of 8 m × 8 m, as illustrated in Figure 4b. The description and geolocation of the botanical classes are provided in Figure 5. Eighty-six and 23 different combinations were obtained in the PG and TG, respectively.

#### 2.2.4. Grouping the Zones and Calculation of the Associated Average Time-budgets

As explained in the previous sections, 576 and 841 zones of 8 m × 8 m were obtained whereas only 86 and 23 different pasture characteristics combinations were identified in the PG and TG, respectively. Consequently, there were many zones characterized by the same combination of pasture characteristics. To reduce this redundancy, we averaged the time-budgets of zones with the same combination, for each cow and for each day of grazing. In this way, we obtained an average time-budget for each of the six behaviors related to each combination of pasture characteristics. The units of these time-budgets (tbu) remained as the seconds per surface unit 8 m × 8 m per cow and per day.

### 2.3. Time-Budget Modeling According to the Pasture Characteristics 

This step aims to model the overall cow location, measured as the amount of time spent per cow per day in each of the 8 m × 8 m areas, and time-budgets, measured as the amount of time spent exhibiting each of the 6 predicted behaviors per cow per day in each of the 8 m × 8 m areas, according to the pasture characteristics. Modeling was carried out for the PG and TG, separately. 

#### 2.3.1. Consideration of the Correlations between the Pasture Characteristics

The lack of independence between some pasture characteristics (e.g., some plant species often found together in the same area) could have biased the interpretation of the linear model results. For this reason, we grouped together characteristics within structural elements, slopes or botanical classes which were highly correlated to reduce the main correlation before applying the linear models. For this, a multiple correspondence analysis was first applied followed by an agglomerative hierarchical clustering (AHC) (aggregation: Ward criterion) using the R package FactoMiner [29]. We identified the most correlated characteristics as those being the most represented within the same cluster (Chi square test: v-test > 2). The groupings carried out are presented in Table 1. 

Subsequent work was carried out to reduce the correlation between the different types of pasture characteristics, such as the correlation between H2 and MS1 (Figure 2a,c). Details of the method used to reduce such correlations are provided in Appendix A (Table A2). The grouping of the pasture characteristics presented in Table 1 remained unchanged.

#### 2.3.2. Modeling with a Linear Mixed Model with an Analysis of Variance 

The effects of the pasture characteristics on the overall cow location and time-budgets were evaluated using 7 different linear mixed models. The reference levels chosen for each characteristic after grouping are presented in Table 1. The two initial models are described in Equations (1) and (2) for the PG and TG, respectively:

(1) $$y_{ijklmnop}\;\sim \;µ+C_{i}+\;D_{j}+T_{k}+\;H_{l}+\;B_{m}+S_{n}+MS_{o}+BC_{p}\;+e_{ijklmnop}\;$$

(2) $$y_{ijklmn}\;\sim \;µ+C_{i}+\;D_{j}+H_{k}+\;B_{l}+S_{m}+BC_{n}\;+e_{ijklmn}\;$$

where the outcome $y_{ijklmnop}\;$and $y_{ijklmn\;}$was either the overall cow location (the total time in seconds spent per cow per day in each of the 8 m × 8 m areas) or the time-budget (the time in seconds spent expressing each of the 6 predicted behaviors per cow per day in each of the 8 m × 8 m areas) for the PG and TG, respectively; µ was the overall mean;$\;C_{i}$ was the cow random effects; $D_{j}$ was the fixed effect of the day of grazing; $H_{l}$ and $H_{k}$ were the fixed effect of hedges for the PG and TG, respectively; $B_{m}$ and $B_{l}$ were the fixed effect of the boundaries for the PG and TG, respectively; $BC_{p}$ and $BC_{n}$ were the fixed effect of the botanical classes for the PG and TG, respectively; $T_{k}$ was the fixed effect of the trees in the PG; $MS_{o}$ was the fixed effect of the soil moisture in the PG and $e_{ijklmnop}$ and $e_{ijklmn}\;$were the random residual effects for the PG and TG, respectively. 

The model parameters were estimated using the R lme4 package [32]. A backward procedure was adopted to get the final model with only significant parameters. An ANOVA of type III was carried out with the R package car [33]. A Tukey test was finally applied to each explanatory parameter to identify the levels which were significantly different from each other. The Tukey test was carried out with the R package emmeans [34].

## 3. Results 

### 3.1. Average Time-Budget of Every Behavior in the Pasture

The dairy cows spent most of their time grazing, representing 56.2% and 71.3% of the time spent in the PG and TG, respectively. The cows also devoted a considerable part of their time to ruminate in lying position (15.9% and 12.1% in the PG and TG, respectively). The rest time in the lying position was about twice as frequent in the PG (13.3%) than in the TG (6.4%). Other behaviors were sparsely represented, leading to a percentage below 6% for both the PG and TG. 

### 3.2. Effect of Each Pasture Characteristic on the Behaviour of Dairy Cows 

The ANOVA results associated with the final models and the adjusted means obtained with the Tukey test for the pasture characteristics in the PG and TG are presented in Table 2 and Table 3, respectively. The ANOVA results associated to the day of the pasture fixed effect are reported in Appendix A (Table A3). The estimates associated to each significant effect in the PG and TG are provided in Appendix A (Table A4 and Table A5). Each effect is reported using the following order: the value of the time-budget (time-budget unit (tbu)—seconds per surface unit of 8 m × 8 m, per cow and per day); the decrease or increase factor of the time-budget associated to the effect in comparison with the basis time-budget, i.e., without any effects (intercept); the significance level associated to the effect. 

#### 3.2.1. Effect of the Pasture Characteristics on the Overall Cow Location and on the Behavior of Dairy Cows in the PG

❖ Overall cow location

All effects for the overall cow location were significant, except the boundaries (Table 2). Cows spent more time under the trees, in particular under “T2” (97.1 tbu; +29.39; *p* < 0.001), in the steep slope areas (12.1 tbu; +2.78; *p* < 0.01), in the “MS2” area (15.8 tbu; +3.9; *p* < 0.05) and in the botanical “Class_3” areas. On the contrary, cows spent less time close to “HN” (−11.0 tbu; −4.45; *p* < 0.01) and in the “MS1” area (−5.1 tbu; −2.59; *p* < 0.05).

❖ Grazing time

All effects on the grazing time were significant (Table 2). The cows spent more time grazing under trees, in particular under “T2” (35.9 tbu; +5.99; *p* < 0.001), close to “H9” (9.4 tbu; +0.8; *p* < 0.05), in steep slope areas (10.9 tbu; +1.12; *p* < 0.001), in the “MS2” area (13.7 tbu; +1.67; *p* < 0.001) and in the botanical “Class_3” areas (12.0 tbu; +1.33; *p* < 0.001). On the contrary, the cows spent less time grazing near “HN” (2.4 tbu; −1.46; *p* < 0.001) and close to “F1” (−1.5 tbu; −1.28; *p* < 0.05).

❖ Walking time

Only the trees and botanical classes significantly affected the walking behavior (*p* < 0.001; Table 2). The cows spent more time walking close to trees, in particular under “T2” (3.0 tbu; +4.55; *p* < 0.001) and close to the botanical “Class_3” areas (1.2 tbu; +11.7; *p* < 0.05).

❖ Ruminating time

Only trees were significant regarding the time spent ruminating while lying (*p* < 0.001; Table 2). The dairy cows spent substantially more time ruminating in lying position close to “T2” (25.8 tbu; +236; *p* < 0.001). Similarly, only trees significantly affected the time spent ruminating in standing position (*p* < 0.001; Table 2.). The dairy cows spent substantially more time ruminating in standing position also close to “T2” (3.4 tbu; +18.15; *p* < 0.001).

❖ Resting time

Only the effect of trees was significant on the rest time while lying (*p* < 0.001; Table 2). In particular, the dairy cows spent substantially more time resting in lying position under “T2” (27.5 tbu; +166.2; *p* < 0.001). Concerning the time spent resting in standing position, the effect was significant for trees (*p* < 0.001; Table 2), hedges (*p* < 0.001; Table 2) and soil moisture (*p* < 0.001; Table 2). The cows spent more time resting while standing under trees, in particular under “T2” (5.1 tbu; +6.06; *p* < 0.001). On the contrary, the cows spent less time resting in the standing position close to “HN” (−0.59 tbu; −1.82; *p* < 0.001) and in the “MS1” area (−0.3 tbu; −1.41; *p* < 0.001). 

#### 3.2.2. Effect of the Pasture Characteristics on the Overall Cow Location and on the Behavior of Dairy Cows in the TG

❖ Overall cow location

All effects are significant for the overall cow location, except for the slope (Table 3). The cows spent more time close to “F4” (19.5 tbu; +0.6; *p* < 0.1) and in botanical “Class_3” areas (18.6 tbu; +0.58; *p* < 0.05). On the contrary, they spent less time near “H2” (1.0 tbu; −0.9; *p* < 0.001) and close to “F1” (6.4 tbu; –0.45; *p* < 0.1).

❖ Grazing time

Hedges (*p* < 0.05; Table 3) and botanical classes *p* < 0.05; Table 3) had a significant effect on the grazing time. Cows spent less time grazing in areas close to “H2” (0.6 tbu; −0.91; *p* < 0.001) and more time grazing in botanical “Class_3” areas (11.7 tbu; + 0.51; *p* < 0.05).

❖ Walking time

Botanical classes had a significant effect on the walking time (*p* < 0.05; Table 3). As for the grazing behavior, cows spent more time walking in botanical “Class_3” (0.93 tbu; +1.06; *p* < 0.01).

❖ Ruminating time

Only botanical classes significantly affected the time spent ruminating in the lying position (*p* < 0.05; Table 3) with more rumination while lying in botanical “Class_3” areas (1.63 tbu; +10.2; *p* < 0.01). The time spent ruminating in the standing position was significantly impacted by the boundaries (*p* < 0.001; Table 3). The cows spent more time ruminating while standing close to “F4” (1.70 tbu; +5.24; *p* < 0.001).

❖ Resting time

No pasture characteristic affected the time spent resting while lying (Table 3). The time spent resting while standing was significantly affected by the boundaries (*p* < 0.01; Table 3). The cows spent more time resting while standing close to “F4” (2.2 tbu; +3.10; *p* < 0.001).

## 4. Discussion

### 4.1. Different Organization of the Dairy Cow Behavior in the Two Pastures 

During the 5 days of grazing, the dairy cows grazed the permanent pasture in the morning and the temporary grassland in the afternoon. The PG involved many natural structural elements, moist areas and a large diversity of plant species dispersed in the field. Conversely, the TG was limited to two hedges and was essentially composed of some major plant species, including ray-grass, bluegrass and brome. The TG was also directly next to the AMS and was directly adjacent to the heifers. A different organization of behaviors of dairy cows was found in these two pastures. A strong organization was observed in the PG around the main pasture characteristics, reflected by some preferred or rejected areas. The dairy cows spent more time under trees, whatever the behavior. They spent more time to graze in the “MS2” area and in the botanical “Class_3” areas, which are mainly constituted by *Holcus lanatus* and *Cerastium* species (Figure 5). In contrast, the “MS1” area was under-visited by dairy cows. The behavior was therefore very structured around the pasture characteristics in this diverse pasture. Conversely, the organization of the dairy cows in the TG was less pronounced. The cows spent more time in the botanical “Class_3” areas (*Sisymbrium officinale* and *Avena fatua;* Figure 5) which were in the AMS access route and close to the fence “F4” which enabled the proximity, sight and socialization with heifers in the neighboring field. Therefore, their behavior seems more influenced by the external environment than by the pasture characteristics themselves in this temporary pasture.

The study protocol involving only two pastures on a single farm for 5 days prevents us from drawing general conclusions on the determinants of the behavior of dairy cows on pasture. However, the organization found under these specific grazing conditions can easily be interpreted in light of the literature. This suggests that, used in contrasted situations chosen on the basis of specific research hypotheses, this approach is promising to explore continuously and automatically the interactions between ruminants and their environment. In the PG, the attractiveness of the trees was similar to the study of Schütz et al. [35], where a high motivation to use shade was reported. It should be noted that the favored tree, “T2”, was the largest tree, providing a large shaded area. This result was also in agreement with Schütz et al. [35] in which cows spent more time in areas with the greatest shade. In this latter study, lying was also observed more frequently in shaded areas than in other places. In our study, the time spent grazing was more important in areas close to trees, as already reported in the literature [36]. The dairy cows spent more time grazing in the “MS2” area, which was located near a natural source of water. As there was no drinking trough in the paddock, the cows may spend more time grazing in “MS2” because of the proximity to the water source. On the contrary, the cows rejected “MS1” although it was a fresh area, probably because they have never suffered from the heat due to the moderate temperatures [17]. The cows also spent more time grazing in botanical “Class_3” areas, which were mainly constituted by the *Holcus lanatus* and *Cerastium* species. This finding corroborates those of O’Donnell and Walton [37], in which free-ranging cattle in an Irish hill-farm spent 81% of their time grazing in spots with *Holcus lanatus*. This comparison should however be more nuanced as the other botanical species in the pasture were different from those of our study. In contrast, the botanical “Class_5”, characterized by a high proportion of *Lolium perenne,* which typically has a higher digestibility and preference than Holcus lanatus, was significantly less grazed by cows. However, Rutter et al. [38] also showed that dairy cows preferentially eat a mixed diet. As *Lolium perenne* was very abundant in the temporary grassland, it is possible that cows grazed other species preferentially in the PG. Finally, it should be mentioned that *Lolium perenne* was not an over-represented species of the botanical “Class_3”, but it still represented 23% of the species in this botanical class (data not shown). In the TG, cows spent more time in the botanical “Class_3” areas while the corresponding vegetation (*Sisymbrium officinale* and *Avena fatua*) is a priori not palatable. This effect is maybe skewed as this botanical class was largely under-represented in the paddock unlike the other two. Furthermore, even if no effect was found for the boundaries close to the AMS (“Bnd_AMS”), the “Class_3” areas were located in the path access of the AMS. The cows may spend more time in these areas simply because of the regular journeys to and from the AMS. This hypothesis also helps to explain why more walking was achieved in these areas. The dairy cows spent more time standing close to the fence “F4” which was directly next to heifers, consistent with the widely reported social and gregarious nature of cattle [39]. Hedges constituted the only shaded areas in the TG, but they were not used by the dairy cows, contrary to what we expected [35]. However, Schütz et al. [35] mentioned that shaded areas are mainly used for resting behaviors, both in standing and lying postures. Therefore, the under-use of the hedges in the TG should be nuanced as the resting behaviors were under-represented in the TG during the afternoon (10.1% of the time corresponding to 19.8 min of resting on average per cow and per day). Moreover, the results obtained during the night on the same paddock highlight a substantial use of hedges for resting behaviors (data not shown). The consistency of the findings obtained by studies on the behavior of dairy cows on pasture thus highlights the potential to combine the predicted behaviors from accelerometer data with positions from GPS data to explore continuously and automatically the resource use by dairy cows at grazing.

### 4.2. Potential of Geolocated Behaviours to Improve Precision Grazing and Animal Health and Welfare

The monitoring of geolocated cow behavior could be used to identify over-grazed locations and then force cows to avoid these areas, as suggested by Laca et al. [40]. As illustrated in this study, dairy cows prefer to graze in specific locations both in heterogeneous [41] and in sown grasslands [42], which may result in a loss of quality forage to less nutritious vegetation in these over-used areas. Adapting pasture management to the use of the resources by the cows could help to prevent an increase in the feeding costs and a decrease in the milk yield related to a depletion of quality forage. Thus, our approach seems particularly relevant in the case of extensive grazing to identify the preferred grazing and resting sites based on grazing management, topography or season, as studied by Feldt and Schlecht [43]. 

Our approach could also be used at the individual level to identify the areas specifically used by each animal and then assess the risk of being affected by certain diseases. For example, the density of the tick *Ixodes ricinus* transmitting *Babesia divergens* is higher in deep hedges [4]. The habitats of the intermediate host snails of *Fasciola hepatica*, responsible for fasciolosis in cattle, are located near water bodies and in soils with poor drainage capacity [5]. Therefore, a fine knowledge of the areas used by each cow would allow a targeted use of drug treatments by treating only animals at risk. 

Identifying the over-visited areas could also decrease the environmental impact at the farm level. Over-frequentation may lead to important amounts of dung on small areas, resulting in a localized release of nitrogen into the soil. A fine knowledge of these spots using our approach could be relevant to adapt fertilization practices. 

Geolocated behaviors are also promising to investigate changes in behavior related to challenging situations [7], including heat stress, physical stress, resource depletion, restricted access to pasture, etc. We were not able to explore such changes in our study because no challenging situations were recorded during the 5 days of experimentation (thermoneutrality, liberal grazing system, etc.), but our methodological framework can be used to explore the changes in both feeding behaviors [44]—static [45] and dynamic [46]—under different stressful situations. Finally, time-budgets were computed in our study but other indicators can be calculated from the predicted behaviors, such as the number of bouts and their average duration. Combined with GPS data, our approach can thus lead to several innovative patterns of behavior, such as the travelled distance during grazing, the duration before lying from the moment of entrance in the paddock, the number of times the areas are visited for grazing as part of the rotation, the resting time close to drinking though and paddock access, etc. The proposed approach therefore offers a range of new behavioral indicators to explore in challenging situations. Such indicators could then be integrated into tools for welfare monitoring. 

### 4.3. Current Technical Limitations

The approach developed in this study has some technical limitations. Currently, the data are stored on a secure digital card and require a manual extraction. An automatic transfer of data would be more convenient for a long-term experiment. The battery life remains the main technical challenge. The battery life of our sensors was only 5 days, which is not enough to explore the relationship between cows and their environment, especially in pastoralism where cows graze continuously over a long period of time. As both the sampling rates of the accelerometer (59.5 Hz) and GPS (1 Hz) sensors are very high, further work is needed to (1) reduce the sampling rate of the accelerometer sensors without decreasing the performance of behavior prediction and to (2) adapt the sampling rate of the GPS sensors to get the desired spatial scale for every application. Another way to deal with this problem is to use solar energy to recharge the battery, as has recently been proposed for virtual fences [47]. Given the potential of the approach developed for research on cattle behavior at grazing, this engineering effort is really worthwhile to make the tool operational for experimental farms.

## 5. Conclusions

The geolocated behavior predicted from the accelerometer and GPS data is a promising way of studying the interactions between cows and their environment. The geolocated behaviors were related to the pasture characteristics, including the vegetation, trees, hedges, soil moisture or external elements of the pasture. In the permanent grassland, the dairy cows spent more time grazing, resting and ruminating near a tree and more time grazing close to a source of water. In the temporary grassland, the behavior of the cows was more influenced by the external environment, such as the presence of heifers or the proximity of the AMS. These findings could be easily related to the literature on dairy cow behavior, suggesting that this approach is promising to explore behaviors in relation to the environment and pasture conditions. Although there are still technical limitations, our approach constitutes a promising way to investigate animal–environment interactions in order to develop precision grazing and improve animal health and welfare.

## Figures and Tables

**Table 1:** Pasture characteristics before and after the grouping carried out to reduce the correlation between characteristics for the permanent grassland and the temporary grassland.

		Permanent Grassland	
		Before Grouping	After Grouping
**Structural Characteristics**	Trees	None	*None*
		T1	T1
		T2	T2
		T3	T3
	Hedges	None	*None*
		H1	H1
		H2, H3, H4	HMS1
		H5	H5
		H6	H6
		H7, H8	Hedges_North noted HN
		H9	H9
		H10	H10
	Boundaries	None	*None*
		PA	PA
		F1	F1
**Slope**		Low	*Low*
		Moderate	Moderate
		Steep	Steep
**Soil Moisture**		Dry	*Dry*
		MS1	MS1
		MS2	MS2
Botanical Classes		Class 1, Class 2	Class_slope
		Class 3	Class 3
		Class 4	*Class 4*
		Class 5	Class 5
		Class 6, 7, 8, 9	Class_Moist_Area noted Class_MA
		Temporary Grassland	
**Structural Characteristics**	Hedges	None	*None*
		H1	H1
		H2	H2
	Boundaries	None	*None*
		F1	F1
		F2, PA	Boundaries_AMS noted Bnd_AMS
		F3	F3
		F4	F4
**Slope**		Low	*Absence*
		Moderate, Steep	Presence
**Botanical Classes**		Class 1	Class 1
		Class 2	*Class 2*
		Class 3	Class 3

Note: All notations referred to Figure 2. Grouped characteristics appear in orange; the reference level for each characteristic is in italic.

**Table 2:** Effect of the pasture characteristics on the overall cow location and time-budget expressed in seconds per surface unit of 8 m × 8 m, per cow and per day in the permanent grassland.

Effect			Overall	Grazing	Walking	Ruminating Lying	Ruminating Standing	Resting Lying	Resting Standing
**Trees**	Sign. ^1^		***	***	***	***	***	***	***
	mean ± se	*None*	20.0 ^a^ ± 3.7	10.6 ^a^ ± 2.3	1.3 ^a^ ± 0.2	1.7 ^a^ ± 0.6	0.4 ^a^ ± 0.2	1.1 ^a^ ± 0.5	0.5 ^a^ ± 0.2
		T1	40.5 ^b^ ± 8.6	14.6 ^ab^ ± 4.6	1.3 ^ab^ ± 0.6	7.4 ^a^ ± 2.6	2.8 ^bc^ ± 0.6	6.6 ^a^ ± 2.6	2.6 ^b^ ± 0.6
		T2	113.9 ^c^ ± 7.5	41.4 ^c^ ± 4.3	3.7 ^c^ ± 0.5	27.6 ^b^ ± 2.1	3.6 ^c^ ± 0.5	28.7 ^b^ ± 2.2	4.9 ^c^ ± 0.5
		T3	36.7 ^b^ ± 6.2	19.1 ^b^ ± 3.7	2.4 ^bc^ ± 0.4	4.6 ^a^ ± 1.7	1.9 ^b^ ± 0.4	2.9 ^a^ ± 1.7	1.5 ^b^ ± 0.4
**Hedges**	Sign. ^1^		*	*	0.68	0.76	0.62	0.96	***
	mean ± se	*None*	54.6 ^b^ ± 4.2	22.3 ^bc^ ± 2.7	2.2 ± 0.3	10.4 ± 1.0	2.2 ± 0.2	9.9 ± 1.0	2.7 ^b^ ± 0.3
		H1	54.6 ^ab^ ± 6.4	23.1 ^bc^ ± 3.8	2.1 ± 0.4	10.9 ± 1.8	2.2 ± 0.4	9.4 ± 1.9	2.7 ^b^ ± 0.4
		H5	56.3 ^ab^ ± 7.7	22.6 ^abc^ ± 4.3	2.5± 0.6	10.6 ± 2.3	2.1 ± 0.5	10.1 ± 2.4	2.6 ^ab^ ± 0.5
		H6	46.2 ^ab^ ± 6.4	18.4 ^ab^ ± 3.7	2.0 ± 0.4	8.9 ± 1.8	1.9 ± 0.4	8.9 ± 1.8	1.8 ^ab^ ± 0.4
		HN	40.4 ^a^ ± 6.5	14.7 ^a^ ± 3.8	1.7 ± 0.4	8.9 ± 1.7	1.8 ± 0.4	8.6 ± 1.7	1.4 ^a^ ± 0.4
		H9	60.3 ^b^ ± 6.5	26.5 ^c^ ± 3.9	2.5 ± 0.5	10.3 ± 1.8	2.6 ± 0.4	10.4 ± 1.9	2.5 ^ab^ ± 0.4
		H10	57.3 ^ab^ ± 6.6	22.4 ^abc^ ± 3.9	2.5 ± 0.5	12.0 ± 1.9	2.1 ± 0.4	9.9 ± 2.0	2.8 ^b^ ± 0.4
**Boundaries**	Sign. ^1^		0.16	***	0.62	0.85	0.80	0.95	0.17
	mean ± se	*None*	49.3 ^a^ ± 5.4	24.6 ^b^ ± 2.5	2.2 ± 0.3	10.4 ± 1.0	2.2 ± 0.2	9.9 ± 1.0	2.2 ± 0.3
		PA	43.0 ^a^ ± 10.4	21.7 ^ab^ ± 4.6	1.7 ± 0.6	9.4 ± 2.8	2.3 ± 0.6	9.6 ± 2.8	2.3 ± 0.7
		F1	37.5 ^a^ ± 9.4	18.0 ^a^ ± 4.1	1.9 ± 0.6	9.4 ± 2.3	1.9 ± 0.5	9.2 ± 2.4	1.4 ± 0.6
**Slope**	Sign. ^1^		**	***	†	0.51	0.99	0.34	0.30
	mean ± se	*Low*	47.9 ^a^ ± 4.7	18.6 ^a^ ± 3.2	1.9 ^a^ ± 0.3	10.2 ± 1.0	2.2 ± 0.2	9.6 ± 1.0	2.4 ± 0.3
		Moderate	53.6 ^ab^ ± 5.4	21.3 ^ab^ ± 3.5	2.1 ^a^ ± 0.3	10.8 ± 1.3	2.2 ± 0.3	10.9 ± 1.3	2.7 ± 0.4
		Steep	56.8 ^b^ ± 5.9	24.4 ^b^ ± 3.6	2.5 ^a^ ± 0.4	11.5 ± 1.5	2.2 ± 0.3	10.8 ± 1.5	2.5 ± 0.4
**Soil moisture**	Sign. ^1^		**	***	0.75	0.20	0.53	0.66	***
	mean ± se	*Dry*	51.3 ^ab^ ± 4.2	19.7 ^a^ ± 2.9	2.2 ± 0.3	10.3 ± 1.0	2.2 ± 0.2	9.9 ± 1.0	2.6 ^b^ ± 0.3
		MS1	43.1 ^a^ ± 6.1	16.2 ^a^ ± 3.8	2.1 ± 0.4	9.2 ± 1.4	2.0 ± 0.3	9.0 ± 1.4	1.5 ^a^ ± 0.4
		MS2	63.9 ^b^ ± 7.1	28.3 ^b^ ± 7.1	2.5 ^a^ ± 0.5	12.6 ± 2.1	2.0 ± 0.4	9.6 ± 2.1	3.0 ^b^ ± 0.5
**Botanical Classes**	Sign. ^1^		*	***	*	0.42	0.27	0.74	†
	mean ± se	*Class 4*	32.6 ^a^ ± 6.6	19.5 ^a^ ± 3.2	2.0 ^ab^ ± 0.3	10.2 ± 1.2	2.0 ± 0.3	9.3 ± 1.2	2.1 ^a^ ± 0.3
		Class 3	42.1 ^b^ ± 7.8	26.3 ^b^ ± 3.7	2.7 ^b^ ± 0.4	11.9 ± 1.5	2.1 ± 0.3	10.7 ± 1.5	2.0 ^a^ ± 0.4
		Class 5	34.5 ^ab^ ± 6.9	18.2 ^a^ ± 3.3	1.7 ^a^ ± 0.3	9.8 ± 1.2	2.5 ± 0.3	10.3 ± 1.2	2.7 ^a^ ± 0.4
		Class_MA	36.7 ^ab^ ± 7.4	21.4 ^ab^ ± 3.5	1.9 ^ab^ ± 0.4	9.1 ± 1.3	2.0 ± 0.3	9.1 ± 1.3	2.7 ^a^ ± 0.4
		Class_slp	33.6 ^ab^ ± 8.6	21.8 ^ab^ ± 4.1	2.5 ^ab^ ± 0.4	10.0 ± 1.7	2.1 ± 0.4	9.8 ± 1.7	2.4 ^a^ ± 0.4

^1^ Significance of each effect: *** *p* < 0.001; ** *p* < 0.01; * *p* < 0.05; † *p* < 0.1. ^a–c^ adjusted means that are different within trees, hedges, boundaries, slope, soil moisture and botanical classes (*p* < 0.05, Tukey’s pairwise comparison). Means are expressed in seconds per surface unit of 8 m × 8 m, per cow and per day (tbu). All notations refer to Table 1.

**Table 3:** Effect of the pasture characteristics on the overall cow location and time-budget expressed in seconds per surface unit of 8 m × 8 m, per cow and per day in the temporary grassland.

Effect			Overall	Grazing	Walking	Ruminating Lying	Ruminating Standing	Resting Lying	Resting Standing
**Hedges**	Sign. ^1^		*	*	0.27	0.37	0.39	0.31	0.22
	mean ± se	*None*	16.8 ^b^ ± 2.2	11.6 ^b^ ± 1.4	0.6 ± 0.1	1.9 ± 0.4	0.7 ± 0.2	0.8 ± 0.2	0.9 ± 0.2
		H1	14.3 ^ab^ ± 3.9	11.4 ^ab^ ± 2.2	0.5 ± 0.2	1.3 ± 0.7	0.8 ± 0.4	0.4 ± 0.3	0.3 ± 0.4
		H2	6.1 ^a^ ± 4.7	4.5 ^a^ ± 2.7	0.1 ± 0.3	0.7 ^a^ ± 0.9	0.2 ± 0.4	0.8 ± 0.2	0.4 ± 0.5
**Boundaries**	Sign. ^1^		*	0.23	0.46	0.22	**	0.24	**
	mean ± se	*None*	12.8 ^ab^ ± 2.1	9.4 ± 1.5	0.5 ± 0.1	1.9 ± 0.4	0.5 ^a^ ± 0.2	0.7 ± 0.2	0.5 ^a^ ± 0.2
		F1	7.5 ^a^ ± 3.4	7.1 ± 2.5	0.4 ± 0.2	0.7 ± 0.6	0.3 ^a^ ± 0.3	0.4 ± 0.3	0.5 ^a^ ± 0.3
		Bnd_AMS	9.9 ^ab^ ± 3.9	7.1 ± 2.9	0.7 ± 0.2	2.1 ± 0.7	0.4 ^a^ ± 0.3	0.8 ± 0.3	0.7 ^a^ ± 0.3
		F3	11.3 ^ab^ ± 5.6	10.1 ± 4.1	0.5 ± 0.4	1.1 ± 1.2	0.6 ^ab^ ± 0.5	0.4 ± 0.6	0.5 ^ab^ ± 0.6
		F4	20.6 ^b^ ± 4.6	13.1 ± 3.3	0.9 ± 0.3	2.8 ± 0.9	1.9 ^b^ ± 0.4	1.5 ± 0.4	2.2 ^b^ ± 0.4
**Slope**	Sign. ^1^		0.96	0.77	0.90	0.94	0.86	0.64	0.62
	mean ± se	*Absence*	12.5 ± 3.0	9.0 ± 1.6	0.5 ± 0.1	1.7 ± 0.4	0.7 ± 0.3	0.8 ± 0.2	2.4 ± 0.3
		Presence	12.6 ± 4.5	9.5 ± 2.0	0.5 ± 0.2	1.7 ± 0.5	0.7 ± 0.2	0.7 ± 0.2	2.7 ± 0.4
**Botanical Classes**	Sign. ^1^		*	*	*	*	0.45	0.57	0.22
	mean ± se	*Class 2*	11.7 ^a^ ± 2.4	9.1 ^ab^ ± 1.4	0.4 ^ab^ ± 0.1	1.2 ± 0.3	0.7 ± 0.1	0.8 ± 0.2	0.8 ± 0.2
		Class 1	6.9 ^a^ ± 4.4	5.2 ^a^ ± 2.7	0.2 ^a^ ± 0.3	1.2 ± 0.8	0.9 ± 0.4	0.4 ± 0.4	0.7 ± 0.5
		Class 3	18.6 ^b^ ± 3.9	13.1 ^b^ ± 2.1	0.9 ^b^ ± 0.2	2.7 ± 0.5	1.0 ± 0.3	0.7 ± 0.3	1.3 ± 0.3

^1^ Significance of each effect: *** *p* < 0.001; ** *p* < 0.01; * *p* < 0.05; † *p* < 0.1. ^a–c^ adjusted means that are different within hedges, boundaries, slope and botanical classes (*p* < 0.05, Tukey’s pairwise comparison). Means are expressed in seconds per surface unit of 8 m × 8 m, per cow and per day (tbu). All notations refer to Table 1.

## Supplementary Materials

The following are available online at https://www.mdpi.com/1424-8220/20/17/4741/s1.

## Abbreviations

The following abbreviations are used in this manuscript:

AMS	Auto Milking System
ANOVA	ANalysis of VAriance
DM	Dry Matter
GPS	Global Positioning System
AHC	Agglomerative Hierarchical Clustering
HMM	Hidden Markov Model
Tbu	Time-budget unit
TG	Temporary Grassland
PG	Permanent Grassland
XGB	eXtreme Gradient Boosting

## Appendix A

**Table A1:** (**a**) Parity and days in the milk of the herd and of the selected cows with (**b**) the associated summary metrics.

**(a)**					
**ID Cow**		**Parity**		**Days in Milk**	
6244		1		33	
6237		1		93	
**6220**		**1**		**111**	
**6196**		**1**		**164**	
**6224**		**1**		**165**	
6178		1		178	
**6219**		**1**		**181**	
**6221**		**1**		**200**	
6214		1		223	
6207		1		252	
**6189**		**1**		**276**	
6193		1		289	
6206		1		300	
6216		1		307	
6203		1		327	
6165		1		329	
6199		1		355	
6186		1		382	
6177		1		432	
6180		1		433	
6158		1		533	
6076		1		590	
**6062**		**1**		**959**	
6190		2		7	
**6099**		**2**		**96**	
6156		2		98	
**6167**		**2**		**135**	
**6166**		**2**		**166**	
**6157**		**2**		**181**	
6159		2		196	
**6130**		**2**		**237**	
**6111**		**2**		**264**	
6100		2		327	
6063		2		332	
6137		2		352	
6122		2		446	
6097		2		455	
6152		3		5	
**5997**		**3**		**43**	
6087		3		89	
5988		3		93	
6074		3		101	
6054		3		103	
**6095**		**3**		**115**	
6092		3		175	
**6050**		**3**		**196**	
**6048**		**3**		**220**	
**6077**		**3**		**220**	
**6089**		**3**		**221**	
6044		3		303	
6007		3		309	
6041		3		313	
6047		3		333	
6035		3		337	
6066		3		366	
6015		3		380	
5958		3		721	
5992		4		5	
6017		4		7	
6005		4		75	
**5981**		**4**		**76**	
**5994**		**4**		**148**	
**5929**		**4**		**155**	
**5947**		**4**		**273**	
5968		4		367	
5887		4		385	
5898		5		166	
**5926**		**5**		**269**	
5798		5		394	
**5819**		**6**		**132**	
**5744**		**7**		**228**	
**(b)**					
		**Mean**	**Standard Deviation**	**Minimum**	**Maximum**
**Herd**	Parity	2.5	1.4	1	7
	Days in milk	250	167	5	959
**Selected cows**	Parity	2.7	1.6	1	7
	Days in milk	209	165	43	959

Note: The cows selected for the experiment are written in bold.

**Table A2:** Re-assignment to prevent the correlation between the different types of pasture characteristics and explanation of the method used.

Combination	Type of Pasture Characteristics	Before Re-Assignment	After Re-Assignment
**Permanent Grassland**			
**1**	Soil moisture	MS1	MS1
	Hedges	Hedges_MS1	*None*
**2**	Slope	Steep	*Low*
	Botanical classes	Class_Slp	Class_Slp
**3**	Botanical classes	Class_3	*Class_4*
	Hedges	H9	H9
**Temporary Grassland**			
**1**	Hedges	H2	H2
	Slope	Presence	*Absence*
	Botanical classes	*Class_2*	*Class_2*
**2**	Boundaries	Bnd_AMS	Bnd_AMS
	Botanical classes	Class_3	*Class_2*
**3**	Boundaries	F3	F3
	Botanical classes	Class_1	*Class_2*
**4**	Boundaries	F4	F4
	Botanical classes	Class 1	*Class_2*

Note: the reference levels are written in italics.

Some different types of pasture characteristics were correlated as H2 and MS1 (Figure 2a,c). All the pasture characteristics concerned by such correlations were also identified using the clusters of the AHC (Section 2.3.1). The correlations between the different types of pasture characteristics led to a repetition of the same information within the same row of the dataset and thus to a potential misinterpretation of the effect of these pasture characteristics. As grouping these characteristics (Table 1) was not possible, we condensed the information on a single characteristic for the entire combination. The characteristics highly correlated within the same combination were either kept to represent the substantial information within the combination, or substituted by the reference level. In this way, the combination of pasture characteristics was summarized by a single characteristic representative of the combination. If the dairy cows spent more time in these zones, then the effect of the characteristic representative of the combination will be significant. However, this process of re-assignment should obviously be considered for the model interpretation. Three and four combinations were concerned in the PG and TG, respectively. The applied re-assignments for these particular combinations are presented in Table A2.

**Table A3:** Effect of the day on the overall cow location and time-budget expressed in seconds per surface unit of 8 m × 8 m, per cow and per day in the permanent grassland and in the temporary grassland.

Effect			Overall	Grazing	Walking	Ruminating Lying	Ruminating Standing	Resting Lying	Resting Standing
**Pasture**	Permanent Grassland								
**Day of Grazing**	Sign. ^1^		*	***	**	**	†	*	0.45
	mean ± se	*Day 1*	45.8 ^a^ ± 5.5	18.4 ^a^ ± 3.5	1.8 ^a^ ± 0.3	8.5 ^a^ ± 1.3	2.0 ^a^ ± 0.3	8.6 ^a^ ± 1.3	1.9 ^a^ ± 0.3
		Day 2	59.4 ^c^ ± 5.5	22.7 ^b^ ± 3.5	1.9 ^a^ ± 0.3	12.8 ^b^ ± 1.3	2.0 ^a^ ± 0.3	13.0 ^b^ ± 1.3	2.0 ^a^ ± 0.3
		Day 3	49.0 ^ab^ ± 5.5	19.1 ^ab^ ± 3.5	2.3 ^ab^ ± 0.3	9.3 ^a^ ± 1.3	2.6 ^a^ ± 0.3	8.7 ^a^ ± 1.3	2.5 ^a^ ± 0.3
		Day 4	52.5 ^abc^ ± 5.5	20.1 ^ab^ ± 3.5	2.8 ^b^ ± 0.3	10.8 ^ab^ ± 1.3	2.1 ^a^ ± 0.3	9.9 ^ab^ ± 1.3	2.0 ^a^ ± 0.3
		Day 5	57.2 ^bc^ ± 5.5	26.9 ^c^ ± 3.5	2.0 ^a^ ± 0.3	10.3 ^ab^ ± 1.3	2.2 ^a^ ± 0.3	9.1 ^a^ ± 1.3	2.2 ^a^ ± 0.3
**Pasture**	Temporary Grassland								
**Day of Grazing**	Sign. ^1^		*	***	0.60	**	*	*	0.21
	mean ± se	*Day 1*	7.6 ^a^ ± 3.6	5.4 ^a^ ± 2.1	0.5 ± 0.2	0.7 ^a^ ± 0.6	0.7 ^a^ ± 0.3	1.2 ^a^ ± 0.3	0.7 ^a^ ± 0.3
		Day 2	16.4 ^bc^ ± 3.6	12.3 ^bc^ ± 2.1	0.6 ± 0.2	1.4 ^ab^ ± 0.6	1.4 ^a^ ± 0.3	0.9 ^a^ ± 0.3	1.2 ^a^ ± 0.3
		Day 3	10.4 ^ab^ ± 3.6	8.2 ^abc^ ± 2.1	0.4 ± 0.2	1.7 ^ab^ ± 0.6	0.4 ^a^ ± 0.3	0.4 ^a^ ± 0.3	0.7 ^a^ ± 0.3
		Day 4	19.1 ^c^ ± 3.6	13.2 ^c^ ± 2.1	0.7 ± 0.2	3.4 ^b^ ± 0.6	0.9 ^a^ ± 0.3	1.0 ^a^ ± 0.3	1.2 ^a^ ± 0.3
		Day 5	8.4 ^ab^ ± 3.6	6.6 ^ab^ ± 2.1	0.4 ± 0.2	1.4 ^a^ ± 0.3	0.4 ^a^ ± 0.3	0.3 ^a^ ± 0.3	0.6 ^a^ ± 0.3

^1^ Significance: *** *p* < 0.001; ** *p* < 0.01; * *p* < 0.05; † *p* < 0.1. ^a–c^ adjusted means that are different in the hedges, boundaries, slope and botanical classes (*p* < 0.05, Tukey’s pairwise comparison). Means are expressed in seconds per surface unit of 8 m × 8 m, per cow and per day (tbu).

**Table A4:** Estimates and significance levels associated with the pasture characteristics and the day of grazing for the permanent grassland.

Effect			Overall	Grazing	Walking	Ruminating Lying	Ruminating Standing	Resting Lying	Resting Standing
**Intercept**		Est. ± se	3.2 ± 4.4	5.1 ± 2.3	0.5 ± 0.3	–0.1 ± 1.0	0.2 ± 0.2	–0.2 ± 1.0	0.7 ± 0.2
		Sign.	0.46	*	†	0.91	0.45	0.86	**
**Day**	Day 2	Est. ± se	1.4 ± 3.3	4.3 ± 1.4	0.2 ± 0.3	4.3 ± 1.2	0.03 ± 0.3	4.4 ± 1.3	
		Sign.	***	**	0.65	***	0.90	***	
	Day 3	Est. ± se	3.2 ± 3.3	0.8 ± 1.4	0.4 ± 0.3	0.8 ± 1.2	0.6 ± 0.3	0.2 ± 1.3	
		Sign.	0.33	0.57	†	0.53	*	0.89	
	Day 4	Est. ± se	6.6 ± 3.3	1.7 ± 1.4	0.9 ± 0.3	2.3 ± 1.2	0.1 ± 0.3	1.3 ± 1.3	
		Sign.	*	0.21	***	†	0.6	0.30	
	Day 5	Est. ± se	1.3 ± 3.3	8.5 ± 1.4	0.2 ± 0.3	1.8 ± 1.2	0.3 ± 0.3	0.5 ± 1.3	
		Sign.	***	***	0.44	0.14	0.3	0.70	
**Trees**	T1	Est. ± se	20.5 ± 7.4	4.0 ± 3.2	0.1 ± 0.5	5.7 ± 2.6	2.4 ± 0.6	5.5 ± 2.7	2.1 ± 0.5
		Sign.	**	0.21	0.91	*	***	*	***
	T2	Est. ± se	93.8 ± 6.1	30.8 ± 2.7	2.5 ± 0.5	26.0 ± 2.1	3.2 ± 0.4	27.7 ± 2.2	4.4 ± 0.4
		Sign.	***	***	***	***	***	***	***
	T3	Est. ± se	16.7 ± 4.9	8.4 ± 2.1	1.1 ± 0.4	2.9 ± 1.7	1.6 ± 0.3	1.8 ± 1.7	1.0 ± 0.3
		Sign.	***	***	**	†	***	0.30	**
**Hedges**	H1	Est. ± se	0.0 ± 4.6	0.8 ± 2.0					0.0 ± 0.3
		Sign.	0.99	0.69					0.95
	H5	Est. ± se	1.7 ± 6.2	0.3 ± 2.7					0.0 ± 0.4
		Sign.	0.78	0.90					0.91
	H6	Est. ± se	–8.5 ± 4.5	–3.9 ± 2.0					0.0 ± 0.3
		Sign.	†	†					0.95
	HN	Est. ± se	–1.4 ± 4.5	–7.5 ± 2.0					–0.9 ± 0.3
		Sign.	**	***					**
	H9	Est. ± se	5.7 ± 4.6	4.3 ± 2.0					–1.3 ± 0.3
		Sign.	0.21	*					***
	H10	Est. ± se	2.4 ± 5.0	0.1 ± 2.2					1.0 ± 0.4
		Sign.	0.21	0.95					0.77
**Boundaries**	PA	Est. ± se		–2.9 ± 3.2					
		Sign.		0.35					
	F1	Est. ± se		–6.6 ± 2.7					
		Sign.		*					
**Slope**	Mod.	Est. ± se	5.6 ± 2.7	2.7 ± 1.2	0.2 ± 0.2				
		Sign.	*	*	0.32				
	Steep	Est. ± se	8.9 ± 3.4	5.7 ± 1.5	0.6 ± 0.2				
		Sign.	**	***	*				
**Soil Moisture**	MS1	Est. ± se	–8.3 ± 3.9	–3.5 ± 1.7					–1.0 ± 0.3
		Sign.	*	*					***
	MS2	Est. ± se	12.6 ± 5.4	8.6 ± 2.4					0.4 ± 0.4
		Sign.	*	***					0.25
**Botanical Classes**	Class 3	Est. ± se	11.8 ± 3.9	6.8 ± 1.7	0.6 ± 0.3				0.0 ± 0.3
		Sign.	**	***	*				0.89
	Class 5	Est. ± se	2.8 ± 3.4	–1.3 ± 1.5	–0.3 ± 0.2				0.6 ± 0.2
		Sign.	0.4	0.36	0.18				*
	Class_MA	Est. ± se	6.6 ± 4.0	1.9 ± 1.8	–0.1 ± 0.2				0.6 ± 0.3
		Sign.	0.1	0.27	0.62				*
	Class_Slp	Est. ± se	5.8 ± 4.5	2.3 ± 1.9	0.4 ± 0.3				0.4 ± 0.3
		Sign.	0.19	0.26	0.21				0.26

Note: Significance of effects: *** *p* < 0.001; ** *p* < 0.01; * *p* < 0.05; † *p* < 0.1.

**Table A5:** Estimates and significance levels associated with the pasture characteristics and the day of grazing for the temporary grassland.

Effect			Overall	Grazing	Walking	Ruminating Lying	Ruminating Standing	Resting Lying	Resting Standing
**Intercept**		Est. ± se	11.7 ± 3.2	7.7 ± 1.9	0.4 ± 0.1	0.1 ± 0.6	0.3 ± 0.3	1.2 ± 0.3	0.5 ± 0.2
		Sign.	***	***	***	0.80	0.32	***	*
**Day**	Day 2	Est. ± se	8.8 ± 3.1	6.9 ± 2.2		0.8 ± 0.7	0.8 ± 0.3	–0.4 ± 0.3	
		Sign.	**	**		0.3	*	0.29	
	Day 3	Est. ± se	2.8 ± 31	2.8 ± 2.2		1.0 ± 0.7	–0.07 ± 0.3	–0.8 ± 0.3	
		Sign.	0.36	0.21		0.2	0.82	*	
	Day 4	Est. ± se	11.5 ± 3.1	7.8 ± 2.2		2.7 ± 0.7	0.4 ± 0.3	–0.3 ± 0.3	
		Sign.	***	***		***	0.22	0.42	
	Day 5	Est. ± se	0.8 ± 3.1	1.2 ± 2.2		0.7 ± 0.7	0.4 ± 0.3	–0.9 ± 0.3	
		Sign.	0.80	0.58		0.33	0.22	*	
**Hedges**	H1	Est. ± se	–2.5 ± 3.5	–0.2 ± 2.2					
		Sign.	0.47	0.94					
	H2	Est. ± se	–10.7 ± 4.0	–7.1 ± 2.5					
		Sign.	**	**					–0.1 ± 0.3
**Boundaries**	F1	Est. ± se	–5.4 ± 3.0				–0.2 ± 0.3		0.80
		Sign.	†				0.53		–0.2 ± 0.3
	Bnd_AMS	Est. ± se	–2.9 ± 3.3				–0.1 ± 0.3		0.59
		Sign.	0.38				0.77		0.0 ± 0.6
	F3	Est. ± se	–1.5 ± 5.2				0.1 ± 0.5		0.94
		Sign.	0.78				0.86		1.7 ± 0.4
	F4	Est. ± se	7.8 ± 4.0				1.4 ± 0.4		***
		Sign.	†				***		
**Slope**	Presence	Est. ± se							
		Sign.							
**Botanical Classes**	Class 1	Est. ± se	–4.8 ± 3.9	–3.8 ± 2.7	–0.2 ± 0.3	0.1 ± 0.8			
		Sign.	0.21	0.14	0.4	0.95			
	Class 3	Est. ± se	6.8 ± 2.9	4.0 ± 1.8	0.5 ± 0.2	1.5 ± 0.6			
		Sign.	*	*	**	**			

Note: Significance of effects: *** *p* < 0.001; ** *p* < 0.01; * *p* < 0.05; † *p* < 0.1.
